# Unpacking uncertainty in polygenic risk scores: Playing “risk score roulette”

**DOI:** 10.1177/13872877261421230

**Published:** 2026-03-23

**Authors:** Louis J. Koizia, Vincent E. S. Allott, Benjamin H. L. Harris

**Affiliations:** 1 Cutrale Perioperative and Ageing Group, Department of Bioengineering, 4615 Imperial College London, London, UK; 2 St. Catherine's College, 6396 University of Oxford, Oxford, UK; 3 Department of Oncology, 6396 University of Oxford, Oxford, UK; 4 Institute for Liver and Digestive Health, Division of Medicine, University College London, London, UK; 5 Department of Psychosocial Rehabilitation, Medical University of Lodz, Lodz, Poland; 6 Collaborating Centre for Values-based Practice, St Catherine's College, Oxford, UK

**Keywords:** Alzheimer’s disease, genetic prediction, polygenic risk scores, reproducibility, risk stratification, uncertainty

Dear Editor,

The recent article by Xue et al. provides an important contribution to the literature on polygenic risk prediction for Alzheimer's disease^1^. Their evaluation of individual-level agreement across multiple polygenic risk score (PRS) methods highlights a critical issue for translation.

Another noteworthy area is the use of random-baseline benchmarking to contextualize PRS performance. Recent work evaluating hypoxia gene signatures has shown how comparing signature summary scores against large numbers of length-matched random signatures reveals the extent to which observed differences reflect true biological signal rather than structural artefact^2^. Applying similar benchmarking in PRS research would provide additional insight and strengthen the robustness of conclusions.

The variation observed across PRS methods also highlights the potential value of developing consensus or ensemble approaches that average across models, reducing the influence of any single algorithm's assumptions. This burgeoning area warrants further consideration and a systematic framework for iterative optimization within the field.

Xue et al. have initiated an essential discussion on PRS reliability. Extending this work to include random-baseline evaluation, clearer distinctions between performance and reliability, and exploration of consensus methods would further advance the robustness and transparency of PRS used in clinical and research settings, ultimately striving for patient benefit.
